# The Last Month of the Century

**Published:** 1900-12

**Authors:** 


### Editorial.

### THE LAST MONTH OF THE CENTURY.

It is well at certain fixed periods in the life of the world to draw aside from the rush of contending elements and stop to consider the past as it affects our several relations socially and professionally. The history of the individual is always of interest to the person who can review his life dispassionately, and to the man of years the thoughts naturally turn to reminiscences of the days lived and the experiences that have made that life interesting to him. The individual that cannot treasure his personal experiences, even though these be marked by many mistakes, is incapable of that feeling that makes of the past a foundation upon which to build for the future. What is true of the individual is more positively true of the world at large and of that nearer, and perhaps dearer, calling which has been to him the support of the life he has lived, and which has brought him not only financial aid, but a personal intercourse with those engaged in the same line of work, and which has made that calling a compensation and a silver lining to the many clouds with which he may have been environed.

When the man of years, in his reminiscent mood, recalls the work done in the past one hundred years, he feels that the progress has been steadily onward. In fact, there is a feeling of self-congratulation that he has been made, actively or passively, a partaker in its progress. It is, perhaps, a trite remark to say that no century can exhibit an equal amount of good work accomplished. This, doubtless, has been said as each century has drawn to its close, and will be said again when those who are now active in this century have ceased from their labors. We, however, can only live our day, and it is no reflection upon those who are able to greet the incoming of the new century that they are able to feel a degree of satisfaction that the nineteenth century has been to them, with all the mistakes made, a brilliant epoch and one worthy to go down into history with an enduring record of great things accomplished.

The inspiriting influence of mental activity that has pervaded all civilizations has had its effect on every branch of human effort. The active medical professional man has lived to see his calling lifted from the empirical stage almost to that of a science. The dentist is closing his century with the happy consciousness that he has passed through the childhood of his profession and is now standing on firm ground awaiting calmly the new day that seems already breaking along the horizon of the coming era.

Dentistry has much to rejoice over. It grovelled in the mire of inexperience in the early days of its life. It tottered in its attempt to walk alongside of the older professions. It became the subject of the sneers and wit of the unthinking. It even failed to secure the honest self-respect of those engaged in it. It had no social life among those who followed it; and up to the middle of the century it was without a character. The last fifty years have made it what it is to-day in all civilized centres of the world. While this is true, it would be unjust to those great names who individually struggled with its difficulties in the old and the new world to say they labored with only partial results; but while honoring these, they were but isolated factors in progress and had but little influence in that great uprising that marked this renascence,—the new birth of dentistry.

In looking back it is interesting to notice the special advances made and the means whereby this progress has been accomplished. In the front rank must be placed organization. The weakness of previous decades was the result of isolated work. The dentist of the earlier periods knew nothing of association, and no advance was made until this was accomplished. We can, therefore, as the century passes into the dim and shadowy history, stop a moment to recall the name of Horace H. Hayden. No monument is erected to the honor of this man, but while dentistry has failed to remember him in this respect, there remains a warm feeling for the one person who never ceased calling the scattered host to gather together until he succeeded in organizing the first national society of dental surgeons.

Has dentistry properly honored the memory of Chapin A. Harris, the man who, when refused recognition by the medical college of his own city, bravely overcame all difficulties and founded the first college of dental surgery? These two men must never be forgotten as the ages roll on. They doubtless builded better than they knew. It is not probable that they could have measured the vast results that followed their immature efforts; but then it is always thus with the little ripples that disturb the great ocean of life. They create no noise, but eventually they are felt in an ever-widening circle.

Organization and education, then, have been the two factors that have made dentistry worthy of our respect and adoration. He is unworthy to be ranked as one of its disciples who fails to feel the enthusiasm of his profession, or who cares only for the loaves and fishes that may have come to him with but little effort on his part.

The day calls up the names of many who have worthily lived their lives and passed on, but their work remains, and it is upon this we have builded. Some of these stand out prominently in the work of the century. Let no one forget the names of Koecker, Fitch, Maynard, Wells, Arthur, Barnum, Garretson, and Bonwill, of this country, Tomes, of England, and Magitot, of France. These are representative of our illustrious dead, and their work will ever remain the glory of their profession. The progress of the century does not, however, lie with these alone, but with those living workers who have added even more to that science that has made dentistry worthy to be classed with the older professions. These will be forced to wait for their apotheoses, but let us never forget the thought that made Wells immortal and that developed the next greatest discovery of the century, the cause of dental caries. The names of Wells, of Hartford, Conn., and Miller, of Berlin, will go down to posterity side by side, the one the apostle of humanity and the other the earnest and successful investigator. The one relieved the surgeon's table of its attendant horrors, and the other gave to dentistry a character for scientific research unequalled in his generation.

The changes that have come to dentistry during the past one hundred years have been heretofore alluded to on these pages. They are part of its history, and familiar to all. The mentality that made these possible is of more importance than the results, valuable and important as these have been. To the mind that can reason from effect back to cause it becomes evident that the mental growth of a people is of more importance in the advancing civilizations than the product of that growth, for it proves that the century has advanced in brain-power, and with this properly conserved there can be no backward steps, but cell to cell there will be continued development and continued progress until the man of to-day will be the pigmy of that future that lies beyond the horizon of the thought of the time.

In bidding a lasting farewell to the century that has bound us in loving bonds, we greet the incoming of the twentieth, and with some measure of prophetic ken can see its possibilities and glory in its yet unborn greatness, its larger measure of life, and its assured professional success.

